# Supplementary material for: Discovering Potential Anti-Oral Squamous Cell Carcinoma Mechanisms from Kochiae Fructus Using Network-Based Pharmacology Analysis and Experimental Validation
Source: Life (Basel). 2023 May 31;13(6):1300. doi: 10.3390/life13061300 (PMC10301113; doi:10.3390/life13061300)
Supplement: Supplementary file 1 [file life-13-01300-s001.zip › Figure S1.pptx]

## Slide 1
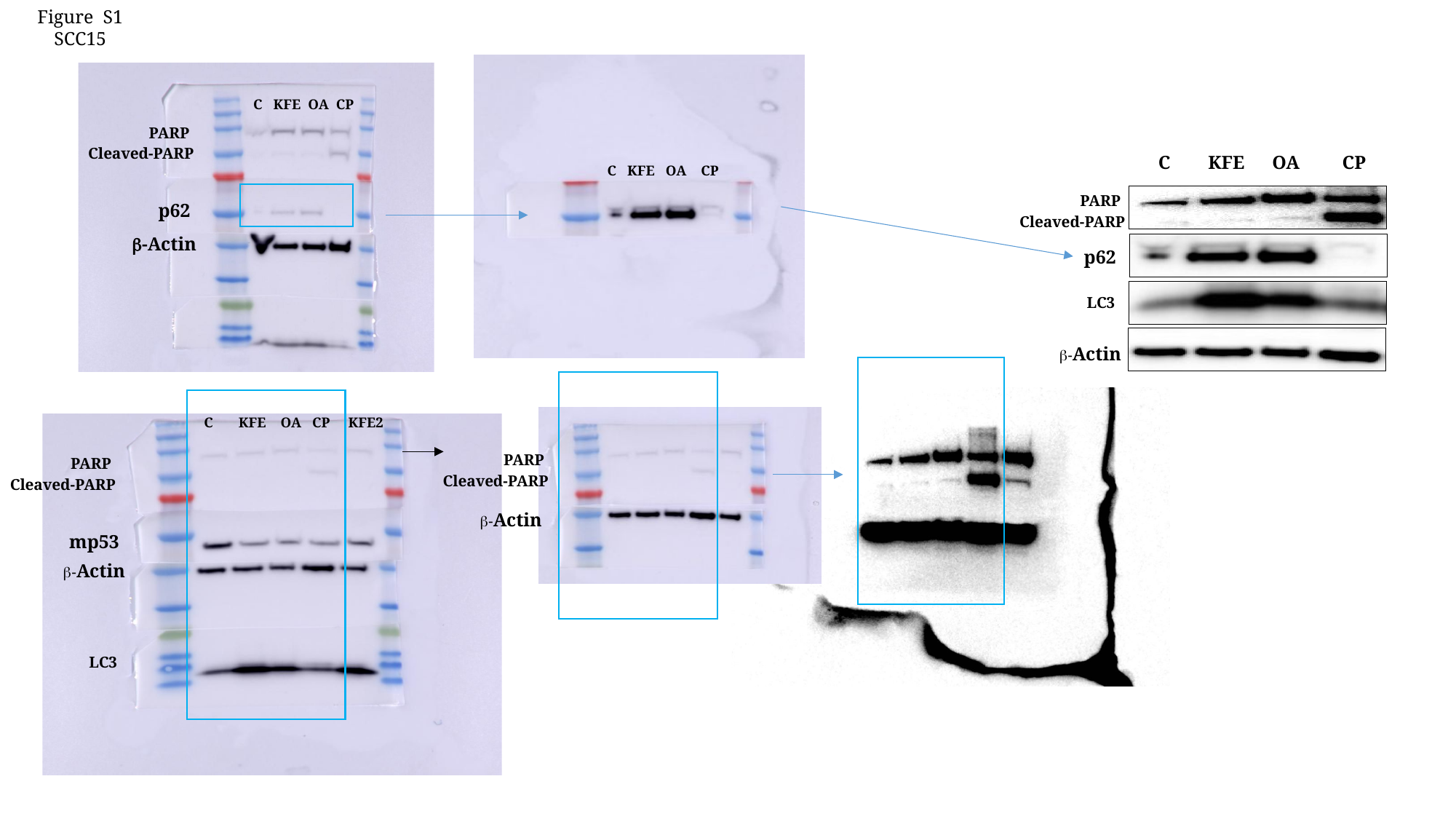

Figure S1
SCC15
C KFE OA CP
PARP
Cleaved-PARP
C KFE	 OA CP
C KFE OA CP
PARP
p62
Cleaved-PARP
β-Actin
p62
LC3
β-Actin
C KFE OA CP KFE2
PARP
PARP
Cleaved-PARP
Cleaved-PARP
β-Actin
mp53
β-Actin
LC3

## Slide 2
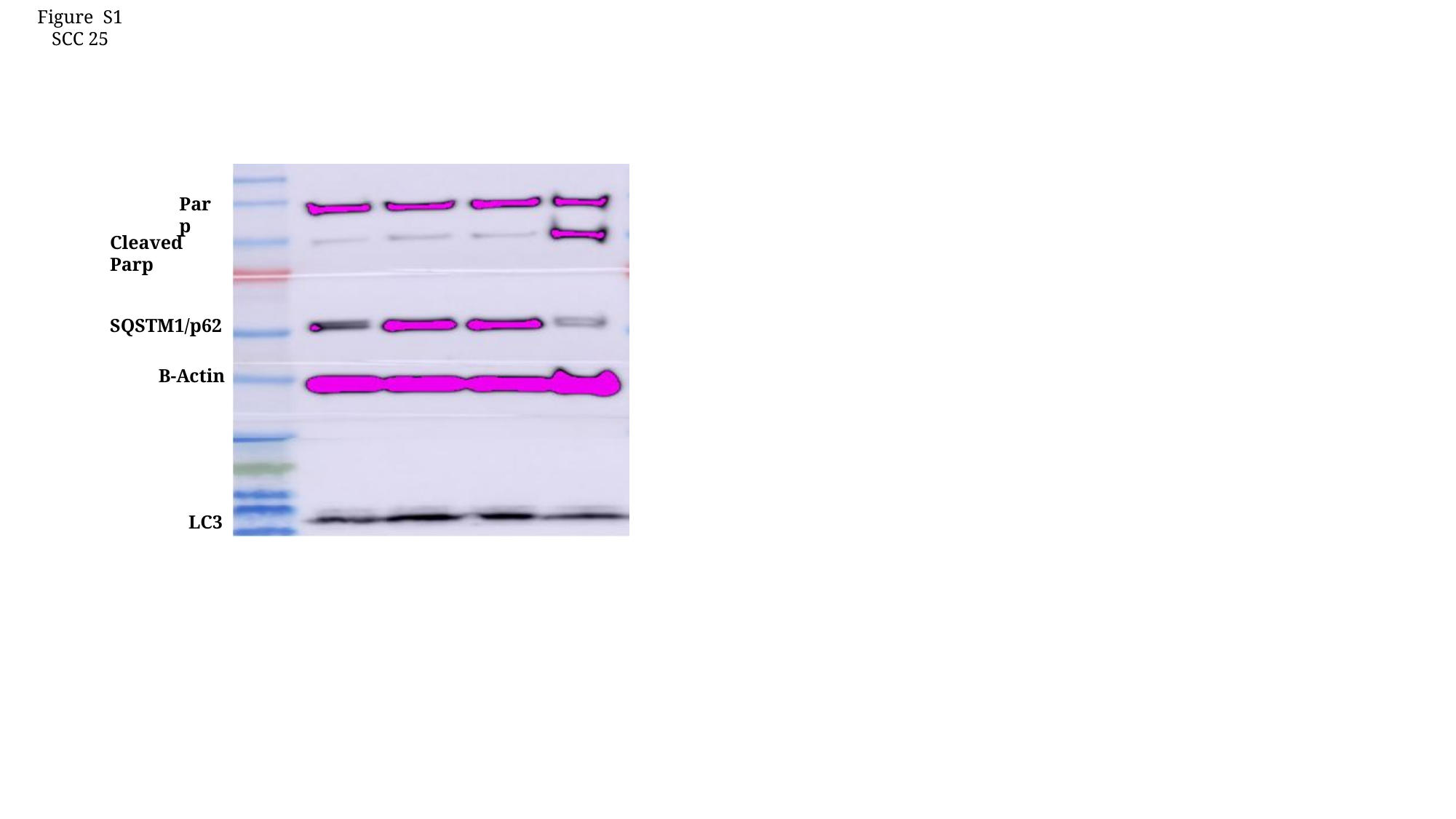

Figure S1
SCC 25
Parp
Cleaved Parp
SQSTM1/p62
Β-Actin
LC3
